# Reasons for non-participation in malformation scans in Denmark: a cohort study

**DOI:** 10.1186/s12884-018-1877-z

**Published:** 2018-06-14

**Authors:** Karina Hjort-Pedersen, Annette Wind Olesen, Ester Garne, Lene Sperling

**Affiliations:** 1 Research Unit of Gynaecology and Obstetrics, Odense University Hospital, University of Southern Denmark, Kloevervaenget 10, 10th floor, 5000 Odense C, Denmark; 2 OPEN, Odense Patient data Explorative Network, Odense University Hospital, Odense, Denmark; 3 Department of Clinical Research, University of Southern Denmark, Odense, Denmark; 4 Department of Gynaecology and Obstetrics, Odense University Hospital, Soendre Blvd., 5000 Odense C, Denmark; 5 Paediatric Department, Lillebaelt Hospital, Kolding, Denmark

**Keywords:** Routine malformation scan, Second trimester, Non-participation, Ultrasound, National screening offer

## Abstract

**Background:**

The aim of the study was to estimate the proportion of women giving birth in two hospitals in the Region of Southern Denmark who did not attend the malformation scan and to elucidate the reasons for not participating.

**Methods:**

In this register-based descriptive study, we used patient administration systems to identify women who had given birth at two Danish hospitals between March 2013 and January 2015. We then linked this information with the hospital database for fetal medicine (Astraia) to identify women who did not attend the malformation scan at week 18–20. We reviewed the medical records of these women to validate the data and to identify the reason for non-participation.

**Results:**

Of 7690 births, 153 (2%) women did not attend the malformation scan. The main reason for non-participation was a passive deselection (81%). Most of these women were not present in Denmark at the time of the malformation scan (61%) and few women declined (8%).

**Conclusions:**

Less than 2% of a birth cohort in two major hospitals in Denmark did not attend the free offer of a malformation scan. Most of these women (81%) did not actively decide against the malformation scan. Very few (0.2%) declined the malformation scan. Non-attendance is not always due to an active decision made by the pregnant woman.

**Electronic supplementary material:**

The online version of this article (10.1186/s12884-018-1877-z) contains supplementary material, which is available to authorized users.

## Background

The malformation scan in the second trimester and the combined first trimester screening (cFTS) are part of the routine antenatal care offered in Denmark and have been offered to all pregnant women since 2006 [1]. Less than 5% of the population does not attend the malformation scan [2] and the reasons for non-participation are unknown.

Denmark has historically had high participation in prenatal diagnostics, even before implementation of the prenatal screening program in 2004. The uptake rate of the malformation scan has increased substantially from 61.6% in 2008 to 95.0% in 2014 [2]. Uptake of the cFTS is similarly high, 93.9% in 2014 [2]. Nearly all (99%) of these scans are performed in public hospitals [2]. Danish studies on attitudes to prenatal testing and abortion have shown that most people in Denmark have a positive attitude to prenatal screening and a gradualistic attitude to termination meaning that people in Denmark have a more liberal attitude to abortion before the fetus becomes viable but are less supportive of terminations late in pregnancy for minor conditions [3, 4]. This could indicate that prenatal screening is quickly accepted in the general population, which is supported by a Dutch study on the populations attitude to prenatal screening [5].

Participation in prenatal screening is high in Denmark compared to other European countries also offering prenatal screening in the first and second trimester. Many studies have investigated factors influencing women’s decision to accept or decline the cFTS [5–11]. Social, religious and cultural factors are suggested to have influence on the uptake rate. A Danish study from 1995 developed a tentative model of these factors suggesting that the factors generally could be divided into societal factors and personal factors. Societal factors were defined as norms and ethics of society for example regarding abortion and disabled people, public media coverage on prenatal screening, attitudes of health services and clinicians, attitudes of other pregnant women, availability of prenatal screening. Personal factors include personal ethics, norms and morality, religious conviction, time in pregnancy, attitude of partner and friends, age, and previous pregnancy experiences [12].

Organization and funding of the healthcare systems seem to be important factors for participation. A study by EUROCAT from 2008 comparing prenatal screening policies in 18 European countries shows that there is a marked difference in prenatal detection rates for Down’s syndrome (DS) and neural tube defects (NTDs) among the countries with and without a national screening program. Detection rates were significantly higher in countries with a national screening offer. However, having a national screening policy did not ensure an equal offer of prenatal screening to all women due to lack of resources, lacking information to the pregnant women or lack of participation. Termination of pregnancy due to a fetal anomaly is legal in most of the European countries, but the legal gestational age limit for termination varies among countries and is a considerable controversy in prenatal screening [13]. The study also shows that even though abortion is legal, uptake rate still differs considerably among these countries suggesting that organizational and cultural factors also influence participation in prenatal screening. In the two countries where abortion was illegal a screening program in the first trimester was not offered. However, women were generally offered an ultrasound examination in the second trimester where the fetus was examined for congenital malformations.

Only a few studies have investigated factors influencing the uptake rate of the malformation scan, and suggest that the factors were slightly different from those related to the cFTS. Age and ethnicity were associated with the cFTS uptake but were not associated with the uptake of the malformation scan [6, 14].

Although there are many similarities between the Danish prenatal screening program and other European programs, it is difficult to compare uptake rates due to differences in health insurance programs, screening policies, abortion legislation and culture.

Our assumption is that very few pregnant women actively decline the routine malformation scan in Denmark and that non-participation is due to other reasons. It is important to investigate the reasons for non-participation to validate the prenatal screening program in order to provide the best prenatal care.

The aim of the study was to estimate the proportion of women giving birth in two hospitals in the Region of Southern Denmark who did not attend the malformation scan and to elucidate the reasons for not participating.

## Methods

### Study design

The study was conducted as a retrospective cohort study of women giving birth at Community Lillebaelt Hospital (LH) and Odense University Hospital (OUH), between March 2013 and December 2015.

### Definition of the malformation scan

The aim of the malformation scan is to detect structural fetal malformations that untreated are associated with a considerable risk of intrauterine death, neonatal death or increased morbidity and mortality in childhood [1]. The malformation scan is performed in accordance with the national guidelines by the Danish Fetal Medicine Society (DFMS) [15]. All sonographers and doctors are certified in accordance with the Fetal Medicine Foundation certification to ensure that the malformation scan is performed uniformly and systematically throughout Denmark [15]. The routine malformation scan is generally performed between gestational week 18 and 20. If the pregnant woman has an obstetric history with fetal malformations or a medical condition with an increased risk of a fetal malformation, she is offered an additional malformation scan around gestational week 16. If specific severe malformations are detected, termination of pregnancy is an option after application.

In Denmark, pregnant women are informed about the possibility of attending the cFTS and the malformation scan at their first visit to the general practitioner. Their decisions are documented in the pregnancy chart sent to the obstetric departments [1].

We extracted data from the electronic record of ultrasound examinations (Astraia), the patient administration system (PAS) and electronic medical records.

### Data sources

Astraia (Astraia software gmbh, version 1.24.7, Germany, https://www.astraia.com/en/) is a clinical ultrasound database used by all public obstetric departments in Denmark as an electronic medical record. Astraia contains data on all ultrasound examinations performed in pregnancy, biochemical data for the cFTS and maternal background information.

PAS manages administrative paperwork in healthcare organizations, mainly hospitals. The essential functions are electronic booking and registration of the patient’s demographics (e.g. name, home address, date of birth) and detailing all patient contacts with the hospital, both outpatient and inpatient. Data from the patient administration system are identical to data reported to the Danish National Patient Registry. Consequently, we consider this source data as valid [16].

We used the medical records to determine the reasons for not attending the malformation scan. Medical records consist of electronic admission notes, progress notes, discharge notes, procedure notes, delivery notes, postpartum notes and out-patient notes.

### Study population

**Fig. 1:**
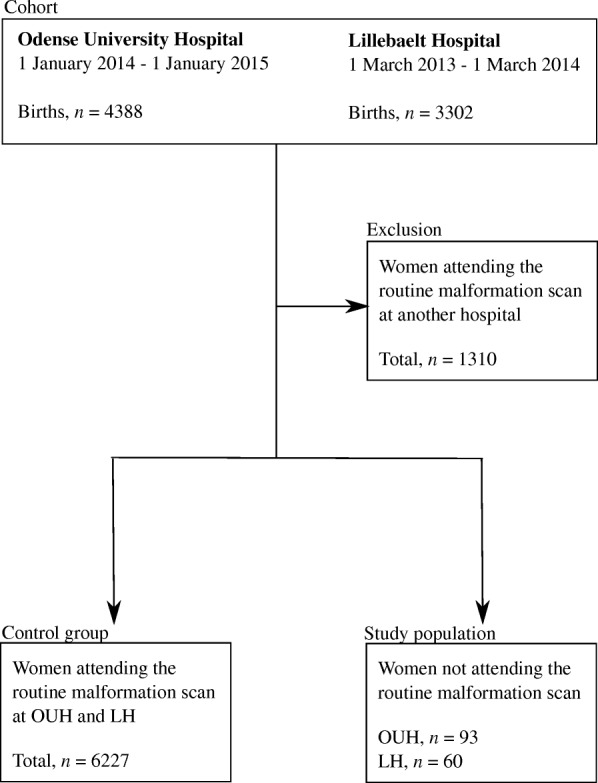
Flowchart study population BMC. Legend: Flowchart showing the identification of women who did not attend the routine malformation scans at Odense University Hospital (OUH) and Lillebaelt Hospital (LH)

We identified women who had given birth in the two hospitals in a two-year period. The study population was pregnant women who did not attend the malformation scan weeks 18–22 from the birth cohort. The women were identified in PAS and Astraia using their civil registration numbers. By linking information from the International Classification of Diseases and Related Health Problems (ICD-10) diagnosis codes DO800-DO848 with ultrasound data from Astraia we were able to identify our study population (Fig. 1). As control group, we used pregnant women from the birth cohort who had attended the routine malformation scan at OUH and LH. Women who had attended the malformation scan at another hospital were not included in the control group (Fig. 1). Medical records were reviewed and validated among women without a registered routine malformation scan to verify data and to identify the reasons for non-participation. Danish experts in fetal medicine accept women who attended the malformation scan at 21 + 0 to 21 + 6 weeks’ gestation as part of the control group even though the examination is performed later than recommended by the national guidelines.

### Variables

Demographic characteristics of the study population included maternal age, body mass index (BMI), country of origin (Denmark/other), native language (Danish/other), civil status (single/ cohabiting), smoking (yes/no), parity (nulliparous/multiparous), first contact to a hospital (date and gestational age), late malformation scan (gestational age) and whether the child had a malformation (yes/no).

Reasons for non-participation in the routine malformation scan were obtained from medical records.

Data were registered in the database Research Electronic Data Capture (REDCap 7.4.23 - © 2018 Vanderbilt university) designed for the study.

### Analysis

A Cubic spline was used to divide the variable years (maternal age) into four age groups and to determine which group should be used as reference (Additional file 1).

Differences between the groups were assessed using χ^2^ tests and Fisher’s exact tests for categorical variables, and independent-samples *t*-tests for continuous variables. A two-sided *p*-value < 0.05 was considered to be statistically significant. Logistic regression was used to calculate odds ratios (OR). REDCap was used as a database for this study and Stata 14 was used for statistical analysis.

### Ethical aspects

The study was conducted in accordance with the regulations of the Committee of Ethics and the use of medical records for the study was approved by the Danish Health Authority.

## Results

The total number of births at LH and OUH was 7690 in the defined study periods. Of these we identified 153 (2.0%) women who did not attend the malformation scan between week 18 + 0 and 21 + 6.

Basic characteristics of the study population are summarized in Table 1.

**Table 1:** Maternal and pregnancy characteristics of participants and non-participants of the malformations scan (gestational week 18–21)

	Non-participants	Participants
Characteristics	N (%)^a^	N (%)^a^
Total	153 (2.4)	6227 (97.6)
Age (years)	27.8 (14–41)	29.5 (16–52)^c^
Smoking status		
Yes	25 (16.3)	325 (10.0)^c^
No	125 (81.7)	5513 (88.5)
Unknown	3 (2.0)	89 (1.4)
Parity		
Nulliparous	82 (53.6)	2621 (42.1)
Multiparous	70 (45.8)	3435 (55.2)^c^
Unknown	1 (0.7)	171 (2.8)
BMI		
< 18.5	10 (6.5)	258 (4.1)
18.5–24.99	85 (55.6)	3522 (56.6)
25.00–29.99	26 (17.0)	1404 (22.6)
≥ 30.00	24 (15.7)	895 (14.4)
Unknown	8 (5.2)	148 (2.4)
Country of origin		
Denmark	58 (37.9)	N/A^b^
Other	93 (60.8)	N/A^b^
Unknown	2 (1.3)	N/A^b^

*BMI* body mass index; ^a^data are given as mean (range) or N (%); ^b^data not available; ^c^*P* <  0.05

In the group of non-participants there were significantly more women who were younger than 25, smoked and were nulliparous. However, after multivariate logistic regression analysis, being younger than 25 was the only significant remaining factor (Table 2). Furthermore, 61% of the non-participants originated from a country other than Denmark.

**Table 2:** Univariable and multivariable logistic regression analyses for prediction of non-participation in the routine malformation scan by maternal characteristics

	Univariable (*n* = 153)		Multivariable (n = 153)	
Variable	OR (95% CI)	*p*-value	OR (95% CI)	*p*-value
Age^a^				
15–19	4.65 (2.28–9.46)	< 0.05	3.51 (1.58–7.81)	0.002
20–24	2.13 (1.46–3.10)	< 0.05	1.70 (1.12–2.57)	0.012
25–39	1.00		1.00	
40–44	0.62 (0.15–2.53)	0.510	0.71 (0.17–2.90)	0.630
Smokingstatus				
No	1.00		1.00	
Yes	1.76 (1.14–2.73)	< 0.05	1.53 (0.97–2.41)	0.069
Parity				
Nulliparous	1.00		1.00	
Multiparous	0.65 (0.03–0.04)	0.009	0.79 (0.55–1.12)	0.180
BMI				
< 18.5	1.61 (0.82–3.13)	0.160	1.31 (0.66–2.59)	0.437
18.5–24.99	1.00		1.00	
≥ 25.00	0.77 (0.49–1.20)	0.240	0.78 (0.50–1.21)	0.270
≥ 30.00	1.11 (0.70–1.76)	0.653	1.09 (0.38–1.72)	0.729

*OR* odds ratio, *CI* confidence interval, *BMI* body mass index; ^a^age groups were generated using a cubic spline (Additional file 1)

In the non-participants group two (1%) fetuses had a congenital malformation. Both malformations were detected prenatally, but after gestational age 21 + 6.

**Fig. 2:**
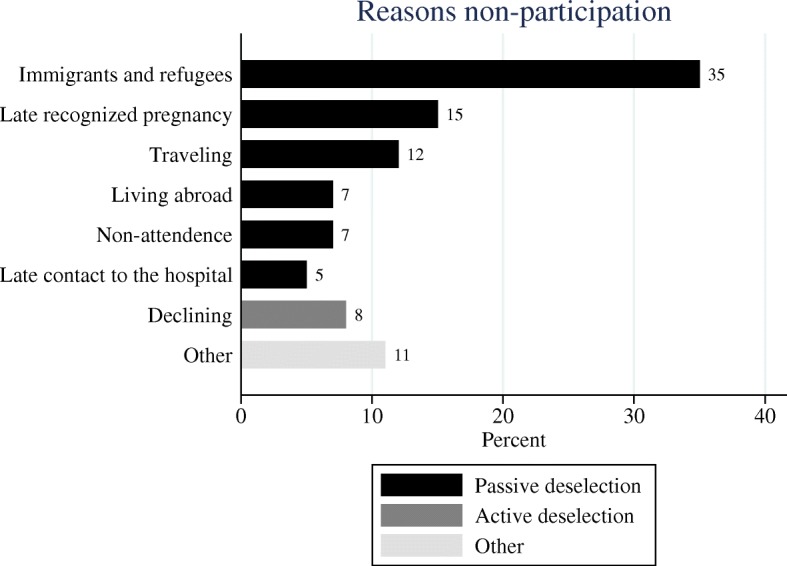
Reasons non-participation BMC. Legend: Bar chart demonstrating the distribution of reasons for non-participation in the routine malformation scans

The majority (81%) of women did not actively decide against the routine malformation scan. There were numerous reasons for non-participation including, women who immigrated to Denmark late in pregnancy, women with late recognized pregnancy, women traveling or living abroad at the time of the routine malformation scan and women who knew they were pregnant yet had no contact to the healthcare system before late in pregnancy (Fig. 2). A common factor for these women is that they were not informed about the possibility of having a malformation scan mainly because they were not present in Denmark. A small proportion (7%) of the women accepted a scheduled appointment for the routine malformation scan but did not show up for the scan. Only 12 (8%) women declined the routine malformation scan which is less than 0.2% of all the women giving birth (12 out of 7690). The category other (11%) contains miscellaneous reasons, i.e. women who declined the malformation scan because they were tourists in Denmark and therefore had to pay. We could not determine the reason of non-participation for 6 (4%) of the women (Fig. 2).

The maternal characteristics of those who declined, and those who did not actively decide against the routine malformation scan are presented in Table 3. In the group of women who did not actively decide against the routine malformation scan there was a significantly larger number of women who spoke another language and were nulliparous.

**Table 3:** Maternal characteristics of women declining the routine malformation scan compared with women passively deselecting the routine malformation scan

	All	Active decliners	Passive decliners
Variable	N (%)^a^	N (%)^a^	N (%)^a^
Total number of women	153	12	124
Age (years)	27.8 (14–41)	30.6 (23–38)	27.5 (14–41)
Civil status			
Single	29 (19.0)	4 (33.3)	33 (26.6)
Cohabiting	123 (80.4)	8 (66.7)	91 (73.4)
Unknown	1 (0.7)	0 (0.0)	0 (0.0)
Smoking status			
Yes	25 (16.3)	4 (33.3)	18 (14.5)
No	125 (81.7)	8 (66.7)	104 (83.9)
Unknown	3 (2.0)	0 (0.0)	2 (1.6)
Parity			
Nulliparous	82 (53.6)	2 (16.7)	75 (60.5)^b^
Multiparous	70 (45.8)	10 (83.3)	49 (39.5)
Unknown	1 (0.7)	0 (0.0)	0 (0.0)
BMI			
< 18.5	10 (6.5)	1 (8.3)	7 (5.9)
18.5–24.99	85 (55.6)	6 (50.0)	71 (60.2)
25.00–29.99	26 (17.0)	2 (16.7)	22 (18.6)
≥ 30.00	24 (15.7)	3 (25.0)	18 (15.3)
Unknown	8 (5.2)	0 (0.0)	6 (4.8)
Country of origin			
Denmark	58 (37.9)	8 (66.7)	48 (38.7)
Other	93 (60.8)	4 (33.3)	75 (60.5)
Unknown	2 (1.3)	0 (0.0)	1 (0.8)
Language			
Danish	77 (50.3)	10 (83.3)	62 (50.0)^b^
Other than Danish	76 (49.7)	2 (16.7)	62 (50.0)
Gestational week first contact hospital	27 + 3	12 + 4	30 + 3
Late malformation scan			
Yes	37 (24.2)	0 (0.0)	33 (26.6)
No	115 (75.2)	12 (100.0)	91 (73.4)
Unknown	1 (0.7)		
Child with a malformation			
Yes	2 (1.3)	0 (0.0)	2 (1.6)
No	150 (98.0)	12 (100.0)	121 (97.6)
Unknown	1 (0.7)	0 (0.0)	1 (0.8)

*BMI* body mass index; ^a^Data are given as mean (range) or N (%). Comparison of active and passive decliners by Fisher’s exact test: ^b^P < 0.05

## Discussion

To our knowledge, this is the first study investigating reasons for non-participation in the routine malformation scan in an unselected cohort of all women giving birth. This descriptive study shows that very few pregnant women did not attend the routine malformation scan (2%) and that the main reason for non-participation was not an active decision against prenatal screening (81%). Most of these women (62%) were not present in Denmark at the time of the malformation scan and 23% of these women had a malformation scan after gestational age 22.

The participation rate in Denmark is high (98%) compared with other European countries offering a similar prenatal screening program (90% in the Netherlands and 93% in Sweden) [6, 14, 17]. Few studies have compared the reasons for non-participation in the malformation scan among countries. A Dutch cohort study based on a questionnaire survey among pregnant women and midwives found that women who identified themselves as religious, were multiparous, had low education or low income were less likely to attend the malformation scan [6]. In our study, we found that women between the ages of 15 and 25 and women originating from a country other than Denmark were less likely to attend the malformation scan. In the group of non-participants 61% were foreigners, this is a considerably large percentage when compared to the general population of the uptake area where the proportion of immigrants is only 13% [18]. This is in line with other studies and with clinical experience suggesting that women declining prenatal screening more often originate from non-western countries [6, 11, 19]. The differences in the finding may be explained by the different study designs. Our study population is based on an unselected cohort of women giving birth, where the Dutch study is based on a cohort of pregnant women attending midwifery practices. Hence, they may have missed information on women who did not attend the midwifery practices before birth.

In the group of non-participants 8% declined the malformations scan. In this group there were significantly more women who were multiparous and who spoke Danish, in comparison to the group who did not actively decide against it. The lack of significance in the analysis of some of the other variables is presumably due to the small numbers.

A Danish nationwide survey from 2016 on characteristics of non-participants of the cFTS found that these women more often originated from a country other than Denmark, spoke another language than Danish, were less well educated and were more religious [11]. The main reason for this difference may be due to different study designs. The survey study had included a random sample of 1000 women who had attended the cFTS and 1000 women who did not. Furthermore, the study suggests that the main reasons for declining were a wish to continue the pregnancy regardless of the test results, probably due to ethical and religious reasons and being opposed to abortion [11]. The proportion of non-participants of the cFTS is slightly higher (6.1%) than non-participants of the routine malformation scan (5%) [2]. We could speculate that reasons for declining the routine malformation in Denmark could be the same reasons as for the cFTS because women who decline the malformation scan often have declined the cFTS too.

Several studies investigating the uptake rate of the cFTS suggest that organization and funding of the healthcare system together with how the prenatal screening policy is presented to the public are important factors for the participation rate [3, 5, 6, 10]. A Dutch study comparing participation rate of the cFTS in the Netherlands, England, and Denmark identified two characteristics in the Dutch screening program that are noticeably different from the screening program in Denmark and may have a considerable influence on the uptake rate. Firstly, there is a charged fee of the cFTS for women under 36 years of age in the Netherlands. The malformation scan is free of charge, but not routinely offered as in Denmark. Secondly, the right not to know seems to be more important for some pregnant women in the Netherlands [5]. This is in line with another Dutch study that states that women’s view on prenatal screening are highly influenced by the social and cultural context in which it is practiced [5, 20].

Sweden has a healthcare system and a national prenatal screening program similar to Denmark. A Swedish study from 2016 shows that the national guidelines on prenatal screening have been interpreted in different ways within the individual counties in Sweden causing the offer of prenatal diagnosis to vary considerably across Sweden, and whilst Denmark has an uptake rate of 95%, the uptake rate of the cFTS in Sweden was only 36.2 and 97% for the routine malformation scan [21, 22]. This suggests that a national screening program does not ensure equal access for all pregnant women if the offer is not identical throughout the country [21]. A Dutch study reflects that the Danish approach could cause the offer to be perceived as a recommendation rather than a choice [5]. However, a Danish study published in 2015 showed that 93% of the women attending the cFTS made an informed choice [23]. We assume that factors contributing to the difference in uptake rate of the cFTS between Denmark and other countries can be applied to the routine malformation scan because pregnant women are informed about both examinations at the same time [1].

Strengths include the use of an unselected cohort of women giving birth contrary to a selected group of women visiting ultrasound units. This has allowed us to investigate the reasons for non-participation and not only reasons for declining. Furthermore, due to the national guidelines on prenatal screening, equal implementation and offer across Denmark, we assume similar results would be obtained throughout Denmark and that our findings cannot be explained by selection bias.

Data were validated by reviewing medical records among all women who had given birth without a registered malformation scan. The study was limited to two hospitals in Denmark which resulted in a low number of non-participants. This may have impacted on the outcome of the present study.

A potential limitation is that the review of the medical records was conducted by one researcher. Possible misinterpretation and misclassification could be reduced by double data entry.

Further, our study population consisted of women giving birth at hospital. We may have missed women giving birth at home (1% of all births in Denmark) and women who had the malformation scan in a private setting (< 1% in Denmark) [2, 24].

Our study showed that the main reason for non-participation was not an active decision against the routine malformation scan. We suggest that a late scan should be offered regardless of the gestational age at the time of referral because a prenatal diagnosis of a malformation may change the management of the pregnancy, delivery and postnatal treatment markedly. It will also give the future parents a chance to prepare themselves for a child with a malformation.

## Conclusion

In this study we found that very few pregnant women (< 2%) did not attend the free offer of a malformation scan in two major hospitals in Denmark. Most of these women (81%) did not make an active decision about attending the malformation scan. More than 60% were not present in Denmark when information about the scan was given. Less than 0.2% declined the scan before week 22. Being younger than 25 or originating from another country was associated with non-attendance. Our findings help to elucidate some of the reasons for non-participation in a country with a national prenatal screening program offered to all pregnant women. Furthermore, that reasons for non-participation may be different from other countries also offering prenatal screening, and non- attendance is not always an active decision made by the pregnant woman.

## Additional file

Additional file 1: Cubic spline. Restricted Cubic spline for the variable years with seven knots (20, 25, 27, 29, 31, 34 and 40) and no other covariate. Exp(xb), Odds ratio; outcome = non-participant. Odds ratio for being a non-participant in correlation with maternal age. (PDF 244 kb)

## Abbreviations

BMI: Body mass index
cFTS: Combined first trimester screening
DFMS: The Danish Fetal Medicine Society
DS: Down syndrome
ICD-10: International Classification of Diseases and Related Health Problems
LH: Lillebaelt Hospital
NTD: Neural tube defect
OR: Odds ratio
OUH: Odense University Hospital
PAS: Patient administration system
