# Supplementary figures and images for: Reasons for non-participation in malformation scans in Denmark: a cohort study
Source: BMC Pregnancy Childbirth. 2018 Jun 14;18:231. doi: 10.1186/s12884-018-1877-z (PMC6001061; doi:10.1186/s12884-018-1877-z)

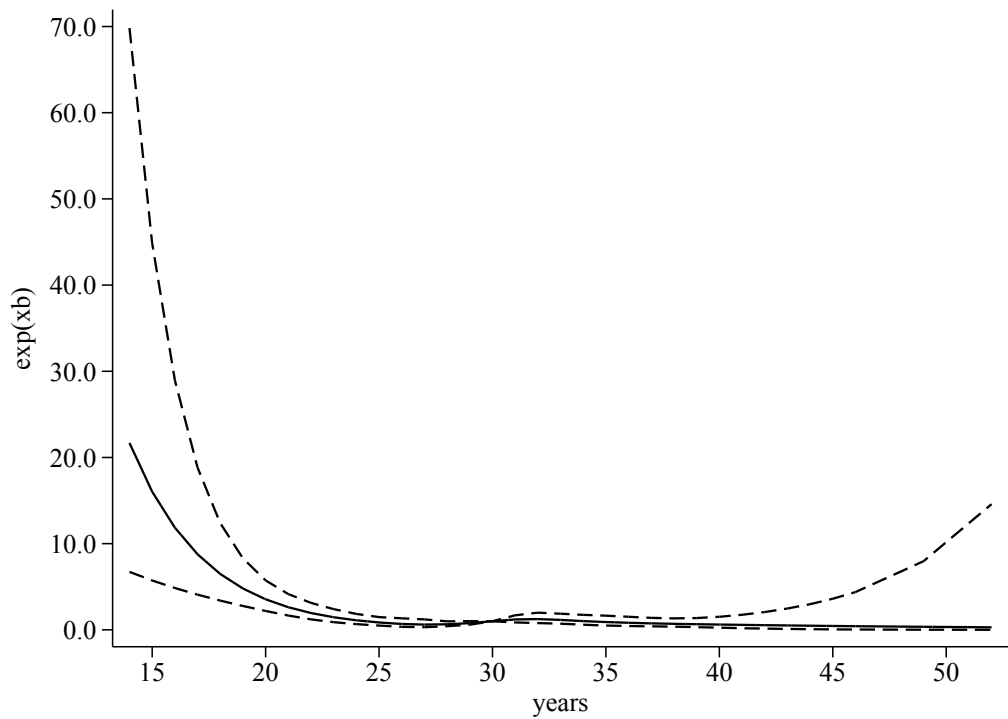

Supplement: Supplementary file 1 — Cubic spline. Restricted Cubic spline for the variable years with seven knots (20, 25, 27, 29, 31, 34 and 40) and no other covariate. Exp(xb), Odds ratio; outcome = non-participant. Odds ratio for being a non-participant in correlation with maternal age. (PDF 244 kb) [file 12884_2018_1877_MOESM1_ESM.pdf]
